# Tetra-μ-aqua-octaaqua­bis(μ-4-chloro­pyridine-2,6-dicarboxyl­ato)bis­(4-chloro­pyridine-2,6-dicarboxyl­ato)tri­cobalt(II)disodium(I) bis­[triaqua­bis(4-chloro­pyridine-2,6-dicarboxyl­ato)cobalt(II)] hexa­hydrate

**DOI:** 10.1107/S1600536807067141

**Published:** 2007-12-21

**Authors:** LaMaryet Moody, Shawna Balof, Shanika Smith, Varma H. Rambaran, Don VanDerveer, Alvin A. Holder

**Affiliations:** a Department of Chemistry and Biochemistry, The University of Southern Mississippi, 118 College Drive #5043, Hattiesburg, MS 39406-0001, USA; b The University of Trinidad and Tobago, O’Meara Campus, Lots 74-98, O’Meara Industrial Park, Arima, Trinidad and Tobago; c Chemistry Department, Clemson University, Clemson SC 29634-0973, USA

## Abstract

The title compound, [Co_3_Na_2_(C_7_H_2_ClNO_4_)_4_(H_2_O)_12_][Co(C_7_H_2_ClNO_4_)(H_2_O)_3_]_2_·6H_2_O, consists of a centrosymmetric dimer of [Co^II^(dipicCl)_2_]^2−^ complex dianions [dipicCl is 4-chloro­pyridine-2,6-dicarboxyl­ate] bridged by an [Na_2_Co^II^(H_2_O)_12_]^4+^ tetra­cationic cluster, two independent [Co(dipicCl)(H_2_O)_3_] complexes, and six water mol­ecules of crystallization. The metals are all six-coordinate with distorted octahedral geometries. The [Co^II^(dipicCl)(H_2_O)_3_] complexes are neutral, with one tridentate ligand and three water molecules. The [Co^II^(dipicCl)_2_]^2−^ complexes each have two tridentate ligands. The [Na_2_Co^II^(H_2_O)_12_]^4+^ cluster has a central Co^II^ ion which is coordinated to six water molecules and lies on a crystallographic inversion center. Four of the water molecules bridge to two sodium ions, each of which have three other water molecules coordinated along with an O atom from the [Co^II^(dipicCl)_2_]^2−^ complex. In the crystal structure, the various units are linked by O—H⋯O hydrogen bonds, forming a three-dimensional network. Two water molecules are disordered equally over two positions.

## Related literature

For related literature, see: Anagnostopoulos (1975 ▶); Cassellato & Vigato (1978 ▶); Chatterjee, Ghosh, Wu & Mak (1998 ▶); Chatterjee, Maji, Ghosh & Mak (1998 ▶); Hartkamp (1962 ▶); Lukes & Jurecek (1948 ▶); Chatterjee *et al.* (1997 ▶); Crans *et al.* (2000 ▶, 2003 ▶, 2006 ▶); D’Ascenzo, Marino, Sabbatini & Bica (1978 ▶); Du *et al.* (2006 ▶); Furst *et al.* (1978 ▶); Ghosh *et al.* (1978 ▶); Lamture *et al.* (1995 ▶); Liu *et al.* (2006 ▶); Su *et al.* (2005 ▶); Yang *et al.* (2002 ▶); Zhou *et al.* (2004 ▶).
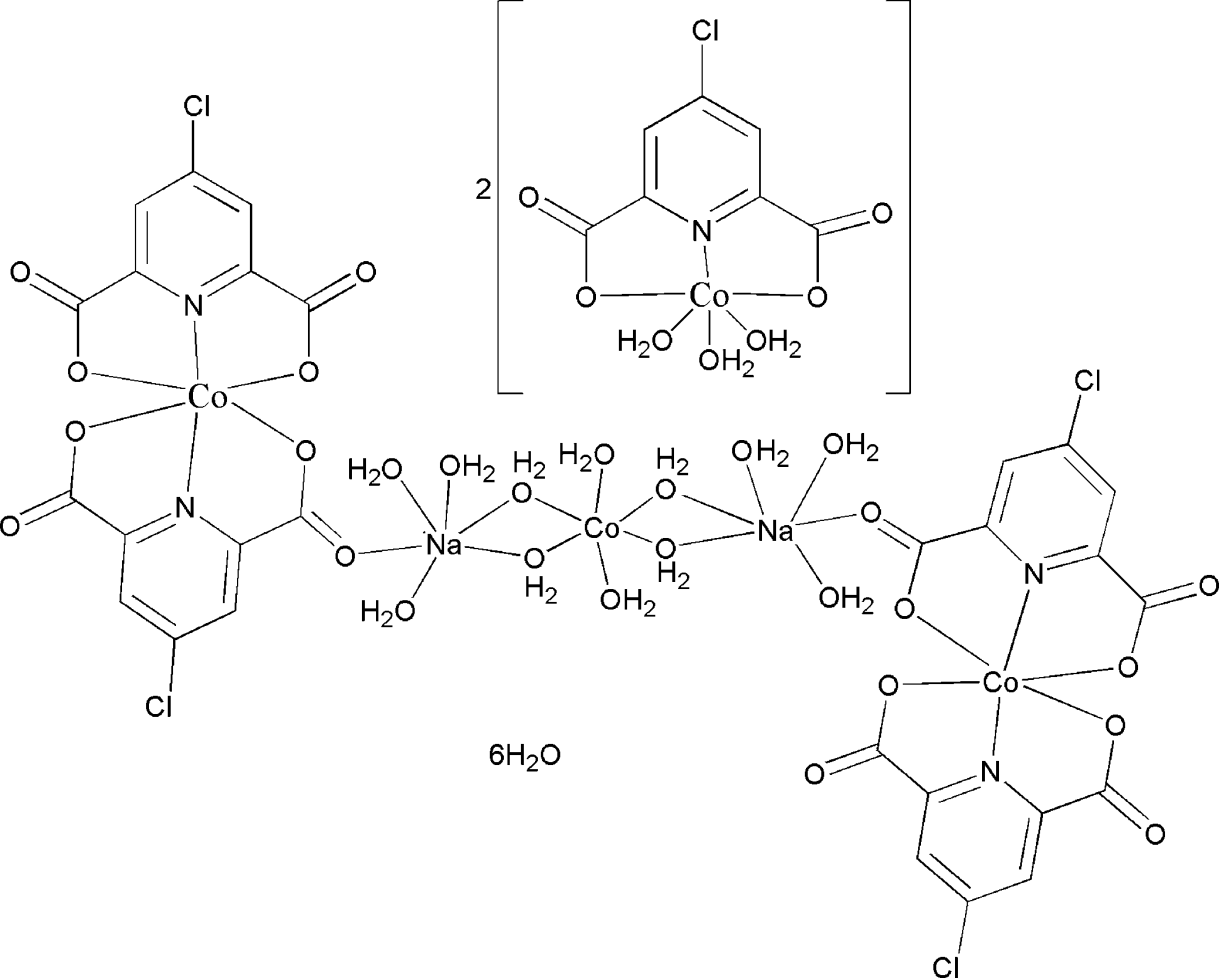


## Experimental

### 

#### Crystal data

[Co_3_Na_2_(C_7_H_2_ClNO_4_)_4_(H_2_O)_12_][Co(C_7_H_2_ClNO_4_)(H_2_O)_3_]_2_·6H_2_O
*M* *_r_* = 1970.29
Triclinic, 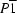

*a* = 9.1539 (18) Å
*b* = 14.475 (3) Å
*c* = 15.476 (3) Å
α = 62.54 (3)°
β = 83.32 (3)°
γ = 80.19 (3)°
*V* = 1791.4 (6) Å^3^
*Z* = 1
Mo *K*α radiation
μ = 1.48 mm^−1^
*T* = 153 (2) K
0.34 × 0.19 × 0.11 mm

#### Data collection

Rigaku Mercury CCD diffractometer
Absorption correction: multi-scan (*REQAB*; Rigaku/MSC 2006 ▶) *T*_min_ = 0.633, *T*_max_ = 0.854
12735 measured reflections
6520 independent reflections
5060 reflections with *I* > 2σ(*I*)
*R*_int_ = 0.024

#### Refinement

*R*[*F* ^2^ > 2σ(*F* ^2^)] = 0.034
*wR*(*F* ^2^) = 0.086
*S* = 1.04
6520 reflections
583 parameters
24 restraints
H atoms treated by a mixture of independent and constrained refinement
Δρ_max_ = 0.58 e Å^−3^
Δρ_min_ = −0.38 e Å^−3^

### 

Data collection: *CrystalClear* (Rigaku/MSC, 2006 ▶); cell refinement: *CrystalClear*; data reduction: *CrystalClear*; program(s) used to solve structure: *SHELXTL* (Bruker, 2000 ▶); program(s) used to refine structure: *SHELXTL*; molecular graphics: *SHELXTL*; software used to prepare material for publication: *SHELXTL*.

## Supplementary Material

Crystal structure: contains datablocks I, global. DOI: 10.1107/S1600536807067141/su2035sup1.cif

Structure factors: contains datablocks I. DOI: 10.1107/S1600536807067141/su2035Isup2.hkl

Additional supplementary materials:  crystallographic information; 3D view; checkCIF report

## Figures and Tables

**Table 1:** Hydrogen-bond geometry (Å, °)

*D*—H⋯*A*	*D*—H	H⋯*A*	*D*⋯*A*	*D*—H⋯*A*
O13—H13*A*⋯O9^i^	0.869 (18)	1.95 (2)	2.778 (3)	160 (3)
O13—H13*B*⋯O4^ii^	0.873 (19)	1.82 (2)	2.687 (3)	174 (3)
O14—H14*A*⋯O11^iii^	0.879 (19)	1.88 (2)	2.747 (3)	171 (4)
O14—H14*B*⋯O7^iv^	0.866 (19)	1.84 (2)	2.688 (3)	167 (4)
O15—H15*A*⋯O24′^v^	0.854 (19)	1.83 (2)	2.650 (6)	161 (4)
O15—H15*A*⋯O24^v^	0.854 (19)	2.13 (2)	2.939 (5)	158 (4)
O15—H15*B*⋯O4^vi^	0.858 (19)	1.885 (19)	2.743 (3)	178 (4)
O16—H16*A*⋯O12	0.884 (19)	1.91 (2)	2.791 (3)	172 (4)
O16—H16*B*⋯O21^vii^	0.833 (19)	1.95 (2)	2.761 (3)	165 (3)
O17—H17*A*⋯O10^v^	0.866 (19)	1.89 (2)	2.752 (3)	170 (3)
O17—H17*B*⋯O20^viii^	0.855 (18)	1.776 (19)	2.628 (3)	174 (4)
O18—H18*A*⋯O19	0.862 (19)	1.89 (2)	2.701 (3)	157 (4)
O18—H18*B*⋯O23′^viii^	0.883 (19)	1.84 (2)	2.703 (7)	165 (4)
O18—H18*B*⋯O23^viii^	0.883 (19)	1.90 (2)	2.753 (6)	162 (3)
O19—H19*B*⋯O6	0.861 (19)	1.89 (3)	2.679 (3)	151 (4)
O20—H20*A*⋯O7^ix^	0.857 (19)	1.89 (2)	2.732 (3)	170 (4)
O21—H21*A*⋯O3^x^	0.862 (19)	1.96 (2)	2.816 (3)	170 (4)
O20—H20*B*⋯O1^x^	0.872 (19)	1.94 (2)	2.802 (3)	171 (4)
O21—H21*B*⋯O12^xi^	0.860 (19)	2.01 (2)	2.837 (3)	161 (4)
O22—H22*A*⋯O7^xii^	0.872 (19)	2.33 (3)	3.136 (4)	154 (4)
O22—H22*B*⋯O21^xiii^	0.87 (2)	2.02 (2)	2.862 (4)	164 (4)
O22—H22*A*⋯O20^xiii^	0.872 (19)	2.69 (5)	3.127 (3)	112 (4)
O23—H23*A*⋯O13^x^	0.872 (19)	1.99 (2)	2.825 (6)	159 (4)
O23′—H23*B*⋯O3^x^	0.97 (3)	2.14 (3)	2.854 (6)	129 (3)
O24—H24*A*⋯O11^xiv^	0.874 (18)	1.95 (2)	2.820 (5)	176 (4)

Symmetry codes: (i) 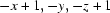; (ii) 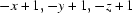; (iii) 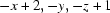; (iv) 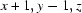; (v) 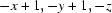; (vi) 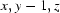; (vii) 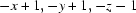; (viii) 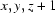; (ix) 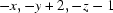; (x) 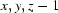; (xi) 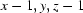; (xii) 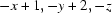; (xiii) 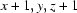; (xiv) 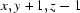.

**Fig. 1.**
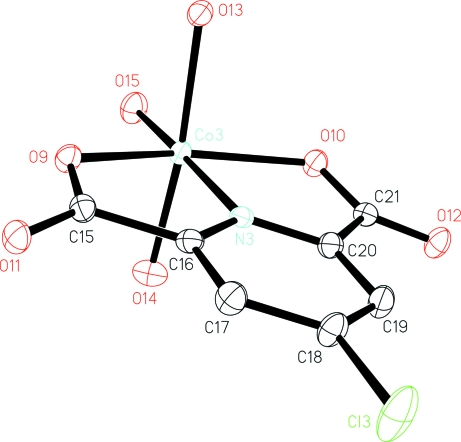
The molecular structure of [Co(dipicCl)(H2O)3], with the thermal ellipsoids drawn at the 50% probability level.

**Fig. 2.**
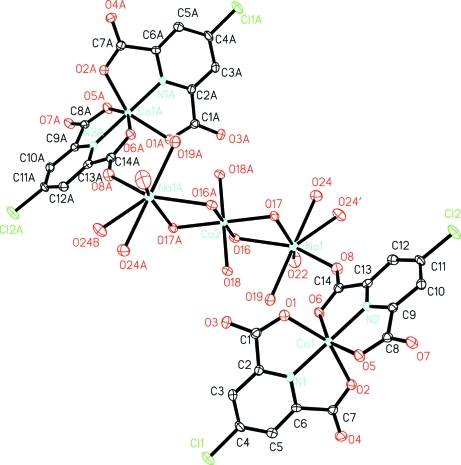
The molecular structure of [Co(dipicCl)2]2. Na2Co(H2O)12, with the thermal ellipsoids drawn at the 50% probability level.

**Fig. 3.**
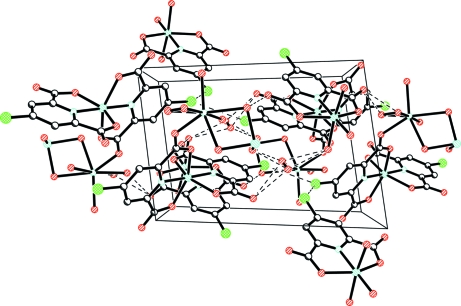
Crystal packing diagram of the title complex viewed along the c axis.

## supplementary crystallographic information

### Comment

Many transition metal complexes involving dipicolinic acid and different cations have been reported (Anagnostopoulos, 1975, Cassellato & Vigato, 1978, D'Ascenzo *et al.*, 1978, Ghosh *et al.*, 1978, Furst *et al.*, 1978). Other examples include metal ions such as chromium (Hartkamp, 1962), copper,(Lukes & Jurecek, 1948), and vanadium (Chatterjee *et al.*, 1997; Chatterjee, Ghosh *et al.*, 1998; Chatterjee, Maji *et al.*, 1998; Crans *et al.*, 2003; Crans *et al.*, 2006, Crans *et al.*, 2000). Examples of cobalt complexes with dipicolinic acid have been reported (Du *et al.*, 2006; Liu *et al.*, 2006; Su *et al.*, 2005; Yang*et al.*, 2002), but none with analogues of dipicolinic acid, except the structure of Co(dipicOH)_3_H_2_O.H_2_O.0.25MeCN reported by (Zhou *et al.*, 2004). As part of our interest in the coordination chemistry of analogues of dipicolinic acid, we now extend this chemistry to include the structural elucidation of the title compound, (I), that was produced in conjunction with an unidentified violet complex.

In compound (I), the cobalt atoms appear in three different coordination environments. These include independent [Co^II^(dipicCl)(H_2_O)_3_] complexes (Fig. 1), and two [Co^II^(dipicCl)_2_]^2-^ complex dianions bridged by a [Na_2_Co^II^(H_2_O)_12_]^4+^ tetra-cationic cluster (Fig. 2). In the latter the central cobalt atom, Co2, occupies a crystallographic inversion center.

In the crystal structure of compound (I) the different complexes and the water molecules of crystallization are linked by O—H···O hydrogen bonds to form a three dimensional network (Fig. 3).

### Experimental

H_2_dipic Cl was synthesized according to the literature procedure (Lamture *et al.*, 1995). [H_2_dipic Cl (4.03 g, 20.0 mmol) was added in small portions to a 100 cm^3^ beaker, which contained a mixture of Na_2_CO_3_ (2.12 g, 20.0 mmol) and warm H_2_O (50 cm^3^). The resulting solution was added dropwise to a stirred solution of CoCl_2_.6H_2_O (4.76 g, 20.0 mmol) in H_2_O (15 cm^3^) over a 30 minute period in a 250 cm^3^ round-bottom flask. The resulting mixture was refluxed for 5 h with stirring. It was then left to stand for 12 h, whereby a violet product formed. The product was filtered off, then washed with water, followed by acetone, and air dried. The filtrate was kept and the mass of the product was recorded. Yield = 3.0 g. This unidentified violet compound is very insoluble in water. The filtrate was allowed to evaporate over six weeks, after which the title complex appeared as brown crystals. For the unidentified violet complex, FT IR (cm^-1^): 3445 (br, *ν*(OH)), 1668 (s, *ν*_as_(CO_2_^-^)), 1615 (very strong, *ν*_as_(CO_2_^-^)),and1388 (s, *ν*(CO_2_^-^)). For [Co(dipic Cl)_2_].Na_2_[Co(dipic Cl)_2_] [Co(dipic Cl)(H_2_O)_3_], FT IR (cm^-1^): 3362 (br, *ν*(OH)), 1614 (very strong, *ν*_as_(CO_2_^-^)),and1373 (s, *ν*(CO_2_^-^)).

### Refinement

Two water molecules (O23 and O24) are each disordered over two positions (O23/O23' and O24/O24') with occupancies of 0.5/0.5. The positions of the water H atoms were located from difference Fourier maps. The O—H distances were restrained to 0.88 (2) Å, with *U*_iso_(H) = 1.5*U*_eq_(O). The remaining H atoms were geometrically placed and treated as riding atoms, with C—H = 0.96Å and *U*_iso_(H) = 1.2*U*_eq_(C).

### Crystal data

**Table:**

[Co_3_Na_2_(C_7_H_2_ClNO_4_)_4_(H_2_O)_12_][Co(C_7_H_2_ClNO_4_)(H_2_O)_3_]_2_·6H_2_O	*Z* = 1
*M**_r_* = 1970.29	*F*_000_ = 997
Triclinic, *P*1	*D*_x_ = 1.826 Mg m^−^^3^
Hall symbol: -P 1	Mo *K*α radiation λ = 0.71073 Å
*a* = 9.1539 (18) Å	Cell parameters from 5409 reflections
*b* = 14.475 (3) Å	θ = 3.7–26.4º
*c* = 15.476 (3) Å	µ = 1.48 mm^−^^1^
α = 62.54 (3)º	*T* = 153 (2) K
β = 83.32 (3)º	Rod, purple
γ = 80.19 (3)º	0.34 × 0.19 × 0.11 mm
*V* = 1791.4 (6) Å^3^	

### Data collection

**Table:**

Rigaku Mercury CCD diffractometer	6520 independent reflections
Radiation source: Sealed Tube	5060 reflections with *I* > 2σ(*I*)
Monochromator: Graphite Monochromator	*R*_int_ = 0.024
Detector resolution: 14.6306 pixels mm^-1^	θ_max_ = 25.5º
*T* = 153(2) K	θ_min_ = 3.2º
ω scans	*h* = −11→11
Absorption correction: multi-scan(REQAB; Rigaku/MSC 2006)	*k* = −15→17
*T*_min_ = 0.633, *T*_max_ = 0.854	*l* = −16→18
12735 measured reflections	

### Refinement

**Table:**

Refinement on *F*^2^	Secondary atom site location: difference Fourier map
Least-squares matrix: full	Hydrogen site location: inferred from neighbouring sites
*R*[*F*^2^ > 2σ(*F*^2^)] = 0.034	H atoms treated by a mixture of independent and constrained refinement
*wR*(*F*^2^) = 0.086	*w* = 1/[σ^2^(*F*_o_^2^) + (0.0467*P*)^2^ + 0.1717*P*] where *P* = (*F*_o_^2^ + 2*F*_c_^2^)/3
*S* = 1.04	(Δ/σ)_max_ = 0.001
6520 reflections	Δρ_max_ = 0.58 e Å^−^^3^
583 parameters	Δρ_min_ = −0.38 e Å^−^^3^
24 restraints	Extinction correction: none
Primary atom site location: structure-invariant direct methods	

### Special details

**Table:**

Geometry. All e.s.d.'s (except the e.s.d. in the dihedral angle between two l.s. planes) are estimated using the full covariance matrix. The cell e.s.d.'s are taken into account individually in the estimation of e.s.d.'s in distances, angles and torsion angles; correlations between e.s.d.'s in cell parameters are only used when they are defined by crystal symmetry. An approximate (isotropic) treatment of cell e.s.d.'s is used for estimating e.s.d.'s involving l.s. planes.
Refinement. Refinement of *F*^2^ against ALL reflections. The weighted *R*-factor *wR* and goodness of fit *S* are based on *F*^2^, conventional *R*-factors *R* are based on *F*, with *F* set to zero for negative *F*^2^. The threshold expression of *F*^2^ > σ(*F*^2^) is used only for calculating *R*-factors(gt) *etc*. and is not relevant to the choice of reflections for refinement. *R*-factors based on *F*^2^ are statistically about twice as large as those based on *F*, and *R*- factors based on ALL data will be even larger.

### Fractional atomic coordinates and isotropic or equivalent isotropic displacement parameters (Å^2^)

**Table:**

	*x*	*y*	*z*	*U*_iso_*/*U*_eq_	Occ. (<1)
Co1	0.27302 (4)	0.83775 (3)	0.19235 (3)	0.01340 (10)	
Cl1	0.34541 (10)	0.46841 (7)	0.63440 (6)	0.0316 (2)	
Cl2	0.21294 (9)	1.22256 (6)	−0.24098 (6)	0.02708 (19)	
N1	0.3011 (3)	0.71984 (18)	0.32895 (18)	0.0138 (5)	
N2	0.2483 (3)	0.95501 (18)	0.05598 (18)	0.0138 (5)	
O1	0.1696 (2)	0.71166 (16)	0.19327 (16)	0.0188 (5)	
O2	0.3910 (2)	0.90072 (16)	0.26077 (16)	0.0186 (5)	
O3	0.1353 (2)	0.54304 (16)	0.28689 (17)	0.0220 (5)	
O4	0.4945 (2)	0.86338 (17)	0.39863 (16)	0.0230 (5)	
O5	0.0749 (2)	0.93466 (16)	0.20562 (16)	0.0194 (5)	
O6	0.4625 (2)	0.80119 (15)	0.11290 (15)	0.0169 (4)	
O7	−0.0654 (2)	1.08848 (16)	0.12304 (16)	0.0203 (5)	
O8	0.5652 (2)	0.85418 (16)	−0.03851 (16)	0.0210 (5)	
C1	0.1770 (3)	0.6265 (2)	0.2730 (2)	0.0163 (6)	
C2	0.2456 (3)	0.6301 (2)	0.3554 (2)	0.0165 (6)	
C3	0.2570 (4)	0.5501 (2)	0.4498 (2)	0.0212 (7)	
H3	0.2162	0.4861	0.4693	0.025*	
C4	0.3296 (3)	0.5654 (2)	0.5155 (2)	0.0203 (7)	
C5	0.3905 (3)	0.6572 (2)	0.4871 (2)	0.0185 (7)	
H5	0.4434	0.6669	0.5317	0.022*	
C6	0.3722 (3)	0.7337 (2)	0.3927 (2)	0.0151 (6)	
C7	0.4242 (3)	0.8415 (2)	0.3475 (2)	0.0156 (6)	
C8	0.0404 (3)	1.0182 (2)	0.1294 (2)	0.0151 (6)	
C9	0.1373 (3)	1.0341 (2)	0.0390 (2)	0.0143 (6)	
C10	0.1218 (3)	1.1194 (2)	−0.0532 (2)	0.0159 (6)	
H10	0.0435	1.1768	−0.0658	0.019*	
C11	0.2245 (3)	1.1174 (2)	−0.1258 (2)	0.0165 (6)	
C12	0.3385 (3)	1.0352 (2)	−0.1082 (2)	0.0165 (6)	
H12	0.4085	1.0340	−0.1590	0.020*	
C13	0.3468 (3)	0.9550 (2)	−0.0140 (2)	0.0144 (6)	
C14	0.4697 (3)	0.8627 (2)	0.0210 (2)	0.0153 (6)	
Co3	0.67155 (4)	0.11941 (3)	0.36043 (3)	0.01259 (10)	
Cl3	1.12718 (10)	0.29703 (7)	0.52031 (6)	0.0343 (2)	
N3	0.8057 (2)	0.17914 (18)	0.41349 (18)	0.0125 (5)	
O9	0.7150 (2)	−0.00397 (15)	0.50786 (15)	0.0156 (4)	
O10	0.6749 (2)	0.27965 (16)	0.24966 (15)	0.0164 (4)	
O11	0.8694 (2)	−0.05463 (16)	0.62997 (16)	0.0185 (5)	
O12	0.8157 (2)	0.41056 (16)	0.19307 (16)	0.0195 (5)	
O13	0.4629 (2)	0.16346 (16)	0.42191 (16)	0.0165 (4)	
H13A	0.395 (3)	0.124 (2)	0.432 (3)	0.025*	
H13B	0.477 (4)	0.159 (3)	0.4786 (17)	0.025*	
O14	0.8596 (2)	0.06266 (18)	0.30523 (17)	0.0208 (5)	
H14A	0.950 (2)	0.061 (3)	0.320 (3)	0.031*	
H14B	0.870 (4)	0.074 (3)	0.2449 (16)	0.031*	
O15	0.5546 (2)	0.06640 (18)	0.29292 (17)	0.0225 (5)	
H15A	0.477 (3)	0.103 (3)	0.263 (3)	0.034*	
H15B	0.538 (4)	0.0025 (17)	0.326 (3)	0.034*	
C15	0.8112 (3)	0.0107 (2)	0.5519 (2)	0.0144 (6)	
C16	0.8614 (3)	0.1193 (2)	0.5014 (2)	0.0135 (6)	
C17	0.9592 (3)	0.1542 (2)	0.5388 (2)	0.0177 (6)	
H17	0.9956	0.1127	0.6031	0.021*	
C18	1.0013 (3)	0.2528 (2)	0.4775 (2)	0.0177 (7)	
C19	0.9472 (3)	0.3142 (2)	0.3847 (2)	0.0181 (7)	
H19	0.9778	0.3818	0.3430	0.022*	
C20	0.8469 (3)	0.2737 (2)	0.3550 (2)	0.0146 (6)	
C21	0.7745 (3)	0.3273 (2)	0.2569 (2)	0.0138 (6)	
Co2	0.5000	0.5000	0.0000	0.01317 (13)	
O16	0.7253 (2)	0.51517 (17)	0.00124 (16)	0.0170 (4)	
H16A	0.758 (4)	0.477 (2)	0.0607 (16)	0.025*	
H16B	0.778 (4)	0.497 (3)	−0.037 (2)	0.025*	
O17	0.4626 (2)	0.66360 (16)	−0.08005 (16)	0.0156 (4)	
H17A	0.424 (4)	0.674 (3)	−0.1328 (18)	0.023*	
H17B	0.394 (3)	0.689 (3)	−0.051 (2)	0.023*	
O18	0.4605 (2)	0.51588 (17)	0.12724 (16)	0.0203 (5)	
H18A	0.528 (3)	0.540 (3)	0.142 (3)	0.030*	
H18B	0.439 (4)	0.464 (2)	0.1846 (18)	0.030*	
Na1	0.69526 (12)	0.70953 (9)	−0.05481 (9)	0.0185 (3)	
O19	0.6582 (3)	0.63560 (18)	0.12553 (18)	0.0242 (5)	
H19A	0.740 (3)	0.621 (3)	0.154 (3)	0.036*	
H19B	0.614 (4)	0.686 (2)	0.137 (3)	0.036*	
O20	0.2393 (2)	0.73840 (18)	−0.99731 (17)	0.0240 (5)	
H20A	0.183 (4)	0.795 (2)	−1.031 (3)	0.036*	
H20B	0.225 (4)	0.724 (3)	−0.9359 (16)	0.036*	
O21	0.0629 (3)	0.52400 (19)	−0.87565 (18)	0.0259 (5)	
H21A	0.083 (4)	0.538 (3)	−0.830 (2)	0.039*	
H21B	0.001 (4)	0.479 (3)	−0.845 (3)	0.039*	
O22	0.9464 (3)	0.6958 (2)	−0.0502 (2)	0.0404 (7)	
H22A	0.994 (5)	0.749 (3)	−0.084 (3)	0.061*	
H22B	0.997 (5)	0.650 (3)	0.000 (2)	0.061*	
O23	0.3333 (6)	0.3650 (4)	−0.7100 (4)	0.0199 (11)	0.50
H23A	0.376 (4)	0.311 (2)	−0.661 (2)	0.030*	
H23B	0.278 (4)	0.398 (3)	−0.680 (3)	0.030*	
O23'	0.3847 (7)	0.3859 (5)	−0.6866 (5)	0.0347 (14)	0.50
O24	0.7164 (5)	0.7828 (4)	−0.2286 (3)	0.0232 (10)	0.50
H24A	0.766 (4)	0.833 (2)	−0.270 (3)	0.035*	
H24B	0.685 (6)	0.826 (4)	−0.203 (4)	0.035*	
O24'	0.7025 (6)	0.8604 (4)	−0.2068 (4)	0.0271 (12)	0.50

### Atomic displacement parameters (Å^2^)

**Table:**

	*U*^11^	*U*^22^	*U*^33^	*U*^12^	*U*^13^	*U*^23^
Co1	0.01611 (19)	0.0112 (2)	0.0106 (2)	−0.00180 (15)	−0.00031 (15)	−0.00306 (16)
Cl1	0.0474 (5)	0.0228 (4)	0.0135 (4)	−0.0051 (4)	−0.0029 (4)	0.0016 (3)
Cl2	0.0288 (4)	0.0243 (4)	0.0135 (4)	−0.0013 (3)	−0.0002 (3)	0.0029 (3)
N1	0.0164 (12)	0.0101 (12)	0.0146 (14)	−0.0010 (9)	0.0002 (10)	−0.0056 (10)
N2	0.0147 (11)	0.0126 (13)	0.0147 (14)	−0.0022 (9)	−0.0016 (10)	−0.0063 (10)
O1	0.0228 (11)	0.0158 (11)	0.0169 (12)	−0.0044 (8)	−0.0016 (9)	−0.0059 (9)
O2	0.0230 (10)	0.0168 (11)	0.0157 (12)	−0.0073 (9)	−0.0009 (9)	−0.0056 (9)
O3	0.0290 (11)	0.0156 (11)	0.0225 (13)	−0.0086 (9)	−0.0014 (10)	−0.0074 (9)
O4	0.0306 (12)	0.0241 (12)	0.0176 (12)	−0.0115 (9)	−0.0010 (10)	−0.0096 (10)
O5	0.0193 (10)	0.0170 (11)	0.0159 (12)	0.0027 (8)	0.0021 (9)	−0.0047 (9)
O6	0.0172 (10)	0.0153 (11)	0.0144 (12)	−0.0004 (8)	0.0011 (8)	−0.0046 (9)
O7	0.0197 (10)	0.0205 (12)	0.0151 (12)	0.0059 (9)	−0.0005 (9)	−0.0062 (9)
O8	0.0194 (10)	0.0206 (12)	0.0209 (13)	−0.0007 (9)	0.0062 (9)	−0.0099 (10)
C1	0.0173 (14)	0.0166 (16)	0.0170 (17)	−0.0027 (12)	0.0009 (12)	−0.0095 (13)
C2	0.0210 (14)	0.0129 (15)	0.0161 (17)	−0.0028 (11)	0.0012 (12)	−0.0073 (12)
C3	0.0330 (17)	0.0128 (16)	0.0166 (17)	−0.0070 (13)	0.0004 (14)	−0.0046 (13)
C4	0.0292 (16)	0.0146 (16)	0.0104 (16)	−0.0008 (12)	0.0014 (13)	−0.0012 (13)
C5	0.0214 (15)	0.0188 (16)	0.0153 (17)	−0.0029 (12)	0.0000 (13)	−0.0078 (13)
C6	0.0184 (14)	0.0167 (16)	0.0112 (15)	−0.0025 (11)	0.0007 (12)	−0.0075 (12)
C7	0.0173 (14)	0.0160 (16)	0.0149 (17)	−0.0039 (11)	0.0032 (12)	−0.0085 (13)
C8	0.0162 (14)	0.0160 (15)	0.0141 (16)	−0.0026 (12)	−0.0018 (12)	−0.0071 (13)
C9	0.0157 (14)	0.0146 (15)	0.0134 (16)	−0.0039 (11)	−0.0012 (12)	−0.0064 (12)
C10	0.0169 (14)	0.0134 (15)	0.0162 (16)	−0.0020 (11)	−0.0013 (12)	−0.0057 (12)
C11	0.0189 (14)	0.0152 (15)	0.0110 (16)	−0.0076 (12)	−0.0027 (12)	0.0001 (12)
C12	0.0153 (14)	0.0197 (16)	0.0144 (16)	−0.0070 (12)	0.0023 (12)	−0.0066 (13)
C13	0.0140 (13)	0.0168 (15)	0.0161 (16)	−0.0036 (11)	0.0006 (12)	−0.0104 (13)
C14	0.0141 (13)	0.0162 (16)	0.0167 (17)	−0.0028 (11)	−0.0008 (12)	−0.0081 (13)
Co3	0.01322 (19)	0.0124 (2)	0.0116 (2)	−0.00269 (14)	−0.00176 (15)	−0.00440 (16)
Cl3	0.0461 (5)	0.0309 (5)	0.0254 (5)	−0.0225 (4)	−0.0155 (4)	−0.0033 (4)
N3	0.0096 (11)	0.0129 (12)	0.0136 (13)	0.0003 (9)	−0.0001 (9)	−0.0055 (10)
O9	0.0152 (10)	0.0157 (11)	0.0147 (11)	−0.0038 (8)	−0.0029 (8)	−0.0047 (9)
O10	0.0165 (10)	0.0153 (11)	0.0153 (11)	−0.0036 (8)	−0.0033 (8)	−0.0040 (9)
O11	0.0189 (10)	0.0166 (11)	0.0148 (12)	−0.0026 (8)	−0.0051 (9)	−0.0016 (9)
O12	0.0219 (10)	0.0173 (11)	0.0140 (12)	−0.0070 (9)	−0.0028 (9)	−0.0007 (9)
O13	0.0151 (10)	0.0163 (11)	0.0160 (12)	−0.0039 (8)	−0.0001 (9)	−0.0050 (9)
O14	0.0167 (10)	0.0305 (13)	0.0165 (12)	0.0007 (9)	−0.0026 (9)	−0.0125 (10)
O15	0.0251 (11)	0.0213 (13)	0.0236 (13)	−0.0082 (10)	−0.0036 (10)	−0.0098 (10)
C15	0.0167 (13)	0.0134 (15)	0.0121 (16)	−0.0013 (11)	0.0009 (12)	−0.0055 (12)
C16	0.0109 (13)	0.0156 (15)	0.0132 (16)	−0.0012 (11)	−0.0008 (11)	−0.0059 (12)
C17	0.0194 (14)	0.0175 (16)	0.0140 (16)	−0.0033 (12)	−0.0036 (12)	−0.0044 (13)
C18	0.0194 (14)	0.0188 (16)	0.0172 (17)	−0.0078 (12)	−0.0032 (12)	−0.0076 (13)
C19	0.0202 (15)	0.0144 (15)	0.0191 (17)	−0.0058 (12)	−0.0017 (13)	−0.0056 (13)
C20	0.0141 (13)	0.0139 (15)	0.0125 (16)	−0.0019 (11)	−0.0001 (11)	−0.0033 (12)
C21	0.0135 (13)	0.0143 (15)	0.0137 (16)	−0.0014 (11)	−0.0005 (12)	−0.0065 (12)
Co2	0.0134 (3)	0.0121 (3)	0.0124 (3)	−0.0013 (2)	−0.0015 (2)	−0.0041 (2)
O16	0.0163 (10)	0.0193 (12)	0.0137 (12)	−0.0009 (8)	−0.0021 (9)	−0.0062 (9)
O17	0.0171 (10)	0.0149 (11)	0.0134 (12)	0.0013 (8)	−0.0037 (9)	−0.0058 (9)
O18	0.0285 (12)	0.0184 (12)	0.0125 (12)	−0.0052 (9)	0.0003 (10)	−0.0054 (9)
Na1	0.0182 (6)	0.0175 (6)	0.0186 (7)	−0.0023 (5)	−0.0006 (5)	−0.0072 (5)
O19	0.0289 (12)	0.0181 (12)	0.0245 (14)	0.0041 (10)	−0.0058 (10)	−0.0101 (10)
O20	0.0261 (12)	0.0253 (13)	0.0157 (12)	0.0068 (9)	−0.0024 (10)	−0.0081 (10)
O21	0.0260 (12)	0.0349 (14)	0.0227 (14)	−0.0129 (10)	0.0017 (10)	−0.0155 (11)
O22	0.0240 (13)	0.0384 (17)	0.0504 (19)	−0.0080 (11)	−0.0110 (12)	−0.0095 (14)
O23	0.023 (3)	0.016 (3)	0.019 (3)	0.003 (2)	−0.001 (2)	−0.008 (2)
O23'	0.035 (3)	0.022 (3)	0.035 (4)	−0.001 (2)	0.011 (3)	−0.006 (3)
O24	0.024 (2)	0.028 (3)	0.018 (3)	−0.012 (2)	0.0065 (19)	−0.009 (2)
O24'	0.027 (3)	0.027 (3)	0.020 (3)	−0.005 (2)	0.005 (2)	−0.006 (2)

### Geometric parameters (Å, °)

**Table:**

Co1—N2	2.016 (3)	O14—H14A	0.879 (19)
Co1—N1	2.024 (3)	O14—H14B	0.866 (19)
Co1—O6	2.143 (2)	O15—H15A	0.854 (19)
Co1—O2	2.147 (2)	O15—H15B	0.858 (19)
Co1—O5	2.147 (2)	C15—C16	1.524 (4)
Co1—O1	2.188 (2)	C16—C17	1.391 (4)
Cl1—C4	1.729 (3)	C17—C18	1.392 (4)
Cl2—C11	1.730 (3)	C17—H17	0.9600
N1—C2	1.341 (4)	C18—C19	1.391 (4)
N1—C6	1.347 (4)	C19—C20	1.388 (4)
N2—C13	1.326 (4)	C19—H19	0.9600
N2—C9	1.341 (4)	C20—C21	1.523 (4)
O1—C1	1.279 (4)	Co2—O18^i^	2.070 (2)
O2—C7	1.255 (4)	Co2—O18	2.070 (2)
O3—C1	1.244 (4)	Co2—O17	2.092 (2)
O4—C7	1.248 (4)	Co2—O17^i^	2.092 (2)
O5—C8	1.263 (4)	Co2—O16^i^	2.114 (2)
O6—C14	1.285 (4)	Co2—O16	2.114 (2)
O7—C8	1.256 (4)	Co2—Na1^i^	3.4979 (15)
O8—C14	1.228 (4)	Co2—Na1	3.4979 (15)
O8—Na1	2.331 (3)	O16—Na1	2.507 (3)
C1—C2	1.511 (4)	O16—H16A	0.884 (19)
C2—C3	1.386 (4)	O16—H16B	0.833 (19)
C3—C4	1.392 (5)	O17—Na1	2.459 (2)
C3—H3	0.9600	O17—H17A	0.866 (19)
C4—C5	1.388 (4)	O17—H17B	0.855 (18)
C5—C6	1.375 (4)	O18—H18A	0.862 (19)
C5—H5	0.9600	O18—H18B	0.883 (19)
C6—C7	1.523 (4)	Na1—O22	2.280 (3)
C8—C9	1.510 (4)	Na1—O24'	2.362 (6)
C9—C10	1.394 (4)	Na1—O24	2.390 (5)
C10—C11	1.386 (4)	Na1—O19	2.490 (3)
C10—H10	0.9600	Na1—H24B	2.13 (6)
C11—C12	1.387 (4)	O19—H19A	0.852 (19)
C12—C13	1.383 (4)	O19—H19B	0.861 (19)
C12—H12	0.9600	O20—H20A	0.857 (19)
C13—C14	1.519 (4)	O20—H20B	0.872 (19)
Co3—O15	2.026 (2)	O21—H21A	0.862 (19)
Co3—N3	2.045 (2)	O21—H21B	0.860 (19)
Co3—O14	2.047 (2)	O22—H22A	0.872 (19)
Co3—O10	2.160 (2)	O22—H22B	0.87 (2)
Co3—O13	2.164 (2)	O23—O23'	0.795 (7)
Co3—O9	2.183 (2)	O23—H23A	0.872 (19)
Cl3—C18	1.732 (3)	O23—H23B	0.877 (19)
N3—C16	1.337 (4)	O23'—H23A	0.98 (3)
N3—C20	1.337 (4)	O23'—H23B	0.97 (3)
O9—C15	1.273 (4)	O24—O24'	1.297 (7)
O10—C21	1.276 (3)	O24—H24A	0.874 (18)
O11—C15	1.250 (4)	O24—H24B	0.88 (2)
O12—C21	1.241 (3)	O24'—H24A	1.27 (4)
O13—H13A	0.869 (18)	O24'—H24B	0.52 (4)
O13—H13B	0.873 (19)		
			
N2—Co1—N1	179.17 (9)	N3—C20—C19	120.9 (3)
N2—Co1—O6	76.81 (9)	N3—C20—C21	113.2 (2)
N1—Co1—O6	102.47 (9)	C19—C20—C21	125.9 (3)
N2—Co1—O2	103.45 (9)	O12—C21—O10	126.6 (3)
N1—Co1—O2	76.18 (9)	O12—C21—C20	118.8 (2)
O6—Co1—O2	95.69 (8)	O10—C21—C20	114.6 (2)
N2—Co1—O5	76.44 (10)	O18^i^—Co2—O18	180.00 (12)
N1—Co1—O5	104.26 (10)	O18^i^—Co2—O17	90.81 (9)
O6—Co1—O5	153.20 (8)	O18—Co2—O17	89.19 (9)
O2—Co1—O5	88.90 (9)	O18^i^—Co2—O17^i^	89.19 (9)
N2—Co1—O1	104.41 (9)	O18—Co2—O17^i^	90.81 (9)
N1—Co1—O1	75.95 (9)	O17—Co2—O17^i^	180.000 (1)
O6—Co1—O1	89.72 (8)	O18^i^—Co2—O16^i^	88.93 (9)
O2—Co1—O1	152.13 (8)	O18—Co2—O16^i^	91.07 (9)
O5—Co1—O1	98.48 (8)	O17—Co2—O16^i^	92.68 (9)
C2—N1—C6	121.0 (3)	O17^i^—Co2—O16^i^	87.32 (9)
C2—N1—Co1	119.6 (2)	O18^i^—Co2—O16	91.07 (9)
C6—N1—Co1	119.39 (19)	O18—Co2—O16	88.93 (9)
C13—N2—C9	121.4 (3)	O17—Co2—O16	87.32 (9)
C13—N2—Co1	119.1 (2)	O17^i^—Co2—O16	92.68 (9)
C9—N2—Co1	119.4 (2)	O16^i^—Co2—O16	180.00 (14)
C1—O1—Co1	115.42 (19)	O18^i^—Co2—Na1^i^	79.08 (7)
C7—O2—Co1	116.71 (18)	O18—Co2—Na1^i^	100.92 (7)
C8—O5—Co1	115.56 (18)	O17—Co2—Na1^i^	136.21 (6)
C14—O6—Co1	115.74 (19)	O17^i^—Co2—Na1^i^	43.79 (6)
C14—O8—Na1	132.80 (19)	O16^i^—Co2—Na1^i^	45.20 (7)
O3—C1—O1	125.9 (3)	O16—Co2—Na1^i^	134.80 (7)
O3—C1—C2	118.8 (3)	O18^i^—Co2—Na1	100.92 (7)
O1—C1—C2	115.2 (2)	O18—Co2—Na1	79.08 (7)
N1—C2—C3	120.8 (3)	O17—Co2—Na1	43.79 (6)
N1—C2—C1	113.5 (3)	O17^i^—Co2—Na1	136.21 (6)
C3—C2—C1	125.7 (3)	O16^i^—Co2—Na1	134.80 (7)
C2—C3—C4	117.9 (3)	O16—Co2—Na1	45.20 (7)
C2—C3—H3	121.1	Na1^i^—Co2—Na1	180.0
C4—C3—H3	121.1	Co2—O16—Na1	98.04 (9)
C5—C4—C3	121.2 (3)	Co2—O16—H16A	110 (2)
C5—C4—Cl1	119.2 (3)	Na1—O16—H16A	114 (2)
C3—C4—Cl1	119.6 (2)	Co2—O16—H16B	111 (3)
C6—C5—C4	117.5 (3)	Na1—O16—H16B	113 (2)
C6—C5—H5	121.2	H16A—O16—H16B	110 (3)
C4—C5—H5	121.2	Co2—O17—Na1	100.14 (9)
N1—C6—C5	121.6 (3)	Co2—O17—H17A	104 (2)
N1—C6—C7	112.0 (3)	Na1—O17—H17A	131 (2)
C5—C6—C7	126.3 (3)	Co2—O17—H17B	110 (2)
O4—C7—O2	126.0 (3)	Na1—O17—H17B	106 (2)
O4—C7—C6	118.4 (3)	H17A—O17—H17B	105 (3)
O2—C7—C6	115.7 (3)	Co2—O18—H18A	116 (3)
O7—C8—O5	125.7 (3)	Co2—O18—H18B	124 (3)
O7—C8—C9	118.1 (3)	H18A—O18—H18B	101 (3)
O5—C8—C9	116.2 (3)	O22—Na1—O8	114.12 (11)
N2—C9—C10	121.0 (3)	O22—Na1—O24'	89.33 (16)
N2—C9—C8	112.2 (3)	O8—Na1—O24'	71.02 (15)
C10—C9—C8	126.8 (3)	O22—Na1—O24	89.85 (14)
C11—C10—C9	116.9 (3)	O8—Na1—O24	99.61 (14)
C11—C10—H10	121.6	O24'—Na1—O24	31.68 (18)
C9—C10—H10	121.6	O22—Na1—O17	155.17 (11)
C10—C11—C12	122.0 (3)	O8—Na1—O17	90.42 (8)
C10—C11—Cl2	118.9 (2)	O24'—Na1—O17	95.61 (15)
C12—C11—Cl2	119.1 (2)	O24—Na1—O17	82.00 (12)
C13—C12—C11	117.0 (3)	O22—Na1—O19	93.27 (11)
C13—C12—H12	121.5	O8—Na1—O19	78.70 (9)
C11—C12—H12	121.5	O24'—Na1—O19	147.79 (15)
N2—C13—C12	121.7 (3)	O24—Na1—O19	176.85 (13)
N2—C13—C14	113.6 (3)	O17—Na1—O19	95.32 (9)
C12—C13—C14	124.6 (3)	O22—Na1—O16	87.95 (10)
O8—C14—O6	126.6 (3)	O8—Na1—O16	147.57 (9)
O8—C14—C13	118.8 (3)	O24'—Na1—O16	135.71 (16)
O6—C14—C13	114.6 (3)	O24—Na1—O16	104.09 (14)
O15—Co3—N3	173.05 (9)	O17—Na1—O16	71.57 (8)
O15—Co3—O14	87.23 (9)	O19—Na1—O16	76.49 (9)
N3—Co3—O14	87.62 (9)	O22—Na1—Co2	124.68 (9)
O15—Co3—O10	99.86 (9)	O8—Na1—Co2	117.26 (6)
N3—Co3—O10	75.88 (9)	O24'—Na1—Co2	125.49 (14)
O14—Co3—O10	94.18 (9)	O24—Na1—Co2	100.42 (12)
O15—Co3—O13	87.85 (9)	O17—Na1—Co2	36.07 (5)
N3—Co3—O13	97.62 (9)	O19—Na1—Co2	78.16 (7)
O14—Co3—O13	173.53 (8)	O16—Na1—Co2	36.76 (5)
O10—Co3—O13	90.79 (9)	O22—Na1—H24B	94.7 (14)
O15—Co3—O9	109.07 (9)	O8—Na1—H24B	78.5 (7)
N3—Co3—O9	75.73 (9)	O24'—Na1—H24B	11.9 (10)
O14—Co3—O9	91.55 (9)	O24—Na1—H24B	21.5 (6)
O10—Co3—O9	150.74 (8)	O17—Na1—H24B	86.3 (14)
O13—Co3—O9	86.10 (9)	O19—Na1—H24B	157.2 (6)
C16—N3—C20	121.5 (3)	O16—Na1—H24B	125.2 (6)
C16—N3—Co3	119.51 (19)	Co2—Na1—H24B	114.0 (12)
C20—N3—Co3	118.6 (2)	Na1—O19—H19A	112 (3)
C15—O9—Co3	115.55 (17)	Na1—O19—H19B	107 (3)
C21—O10—Co3	115.63 (18)	H19A—O19—H19B	102 (4)
Co3—O13—H13A	114 (2)	H20A—O20—H20B	109 (4)
Co3—O13—H13B	110 (2)	H21A—O21—H21B	100 (4)
H13A—O13—H13B	106 (3)	Na1—O22—H22A	122 (3)
Co3—O14—H14A	124 (3)	Na1—O22—H22B	123 (3)
Co3—O14—H14B	122 (3)	H22A—O22—H22B	110 (4)
H14A—O14—H14B	104 (3)	O23'—O23—H23A	72 (3)
Co3—O15—H15A	121 (3)	O23'—O23—H23B	70 (3)
Co3—O15—H15B	115 (3)	H23A—O23—H23B	101 (4)
H15A—O15—H15B	109 (4)	O23—O23'—H23A	57.8 (18)
O11—C15—O9	126.9 (3)	O23—O23'—H23B	58.8 (18)
O11—C15—C16	117.6 (3)	H23A—O23'—H23B	88 (3)
O9—C15—C16	115.4 (3)	O24'—O24—Na1	73.0 (3)
N3—C16—C17	121.8 (3)	O24'—O24—H24A	68 (3)
N3—C16—C15	113.0 (2)	Na1—O24—H24A	129 (3)
C17—C16—C15	125.2 (3)	O24'—O24—H24B	17 (3)
C16—C17—C18	116.2 (3)	Na1—O24—H24B	62 (4)
C16—C17—H17	121.9	H24A—O24—H24B	85 (4)
C18—C17—H17	121.9	O24—O24'—Na1	75.3 (3)
C19—C18—C17	122.3 (3)	O24—O24'—H24A	39.8 (10)
C19—C18—Cl3	120.3 (2)	Na1—O24'—H24A	108.9 (13)
C17—C18—Cl3	117.4 (2)	O24—O24'—H24B	29 (4)
C20—C19—C18	117.2 (3)	Na1—O24'—H24B	58 (7)
C20—C19—H19	121.4	H24A—O24'—H24B	69 (5)
C18—C19—H19	121.4		

Symmetry codes: (i) −*x*+1, −*y*+1, −*z*.

### Hydrogen-bond geometry (Å, °)

**Table:**

*D*—H···*A*	*D*—H	H···*A*	*D*···*A*	*D*—H···*A*
O13—H13A···O9^ii^	0.869 (18)	1.95 (2)	2.778 (3)	160 (3)
O13—H13B···O4^iii^	0.873 (19)	1.82 (2)	2.687 (3)	174 (3)
O14—H14A···O11^iv^	0.879 (19)	1.88 (2)	2.747 (3)	171 (4)
O14—H14B···O7^v^	0.866 (19)	1.84 (2)	2.688 (3)	167 (4)
O15—H15A···O24'^i^	0.854 (19)	1.83 (2)	2.650 (6)	161 (4)
O15—H15A···O24^i^	0.854 (19)	2.13 (2)	2.939 (5)	158 (4)
O15—H15B···O4^vi^	0.858 (19)	1.885 (19)	2.743 (3)	178 (4)
O16—H16A···O12	0.884 (19)	1.91 (2)	2.791 (3)	172 (4)
O16—H16B···O21^vii^	0.833 (19)	1.95 (2)	2.761 (3)	165 (3)
O17—H17A···O10^i^	0.866 (19)	1.89 (2)	2.752 (3)	170 (3)
O17—H17B···O20^viii^	0.855 (18)	1.776 (19)	2.628 (3)	174 (4)
O18—H18A···O19	0.862 (19)	1.89 (2)	2.701 (3)	157 (4)
O18—H18B···O23'^viii^	0.883 (19)	1.84 (2)	2.703 (7)	165 (4)
O18—H18B···O23^viii^	0.883 (19)	1.90 (2)	2.753 (6)	162 (3)
O19—H19B···O6	0.861 (19)	1.89 (3)	2.679 (3)	151 (4)
O20—H20A···O7^ix^	0.857 (19)	1.89 (2)	2.732 (3)	170 (4)
O21—H21A···O3^x^	0.862 (19)	1.96 (2)	2.816 (3)	170 (4)
O20—H20B···O1^x^	0.872 (19)	1.94 (2)	2.802 (3)	171 (4)
O21—H21B···O12^xi^	0.860 (19)	2.01 (2)	2.837 (3)	161 (4)
O22—H22A···O7^xii^	0.872 (19)	2.33 (3)	3.136 (4)	154 (4)
O22—H22B···O21^xiii^	0.87 (2)	2.02 (2)	2.862 (4)	164 (4)
O22—H22A···O20^xiii^	0.872 (19)	2.69 (5)	3.127 (3)	112 (4)
O23—H23A···O13^x^	0.872 (19)	1.99 (2)	2.825 (6)	159 (4)
O23'—H23B···O3^x^	0.97 (3)	2.14 (3)	2.854 (6)	129 (3)
O24—H24A···O11^xiv^	0.874 (18)	1.95 (2)	2.820 (5)	176 (4)

Symmetry codes: (ii) −*x*+1, −*y*, −*z*+1; (iii) −*x*+1, −*y*+1, −*z*+1; (iv) −*x*+2, −*y*, −*z*+1; (v) *x*+1, *y*−1, *z*; (i) −*x*+1, −*y*+1, −*z*; (vi) *x*, *y*−1, *z*; (vii) −*x*+1, −*y*+1, −*z*−1; (viii) *x*, *y*, *z*+1; (ix) −*x*, −*y*+2, −*z*−1; (x) *x*, *y*, *z*−1; (xi) *x*−1, *y*, *z*−1; (xii) −*x*+1, −*y*+2, −*z*; (xiii) *x*+1, *y*, *z*+1; (xiv) *x*, *y*+1, *z*−1.

## References

[bb1] Anagnostopoulos, A. (1975). *J. Coord. Chem.* **4**, 231–233.

[bb2] Bruker (2000). *SHELXTL* Version 6.10. Bruker AXS Inc., Madison, Wisconsin, USA.

[bb3] Cassellato, U. & Vigato, P. A. (1978). *Coord. Chem. Rev.* **26**, 85–159.

[bb4] Chatterjee, M., Ghosh, S. & Nandi, A. K. (1997). *Polyhedron*, **16**, 2917–2923.

[bb5] Chatterjee, M., Ghosh, S., Wu, B.-M. & Mak, T. C. W. (1998). *Polyhedron*, **17**, 1369–1374.

[bb6] Chatterjee, M., Maji, M., Ghosh, S. & Mak, T. C. W. (1998). *J. Chem. Soc. Dalton Trans.* pp. 3641–3646.

[bb7] Crans, D. C., Mahroof-Tahir, M., Johnson, M. D., Wilkins, P. C., Yang, L., Robbins, K., Johnson, A., Alfano, J. A., Godzala, M. E., Austin, L. T. & Willsky, G. R. (2003). *Inorg. Chim. Acta*, **356**, 365–378.

[bb8] Crans, D. C., Rithner, C. D., Baruah, B., Gourley, B. L. & Levinger, N. E. (2006). *J. Am. Chem. Soc.* **128**, 4437–4445. 10.1021/ja0583721 16569021

[bb9] Crans, D. C., Yang, L., Jakusch, T. & Kiss, T. (2000). *Inorg. Chem.* **39**, 4409–4416.

[bb10] D’Ascenzo, G., Marino, A., Sabbatini, M. & Bica, T. (1978). *Thermochim. Acta*, **25**, 325–332.

[bb11] Du, M., Cai, H. & Zhao, X.-J. (2006). *Inorg. Chim. Acta*, **359**, 673–679.

[bb12] Furst, W., Gouzerch, P. & Jeannin, Y. (1978). *J. Coord. Chem.* **8**, 237–243.

[bb13] Ghosh, S., Banerjee, T. K. & Ray, P. K. (1978). *J. Indian Chem. Soc.* **55**, 610–611.

[bb14] Hartkamp, H. (1962). *Z. Anal. Chem.* **187**, 16–29.

[bb15] Lamture, J. B., Zhou, Z. H., Kumar, A. S. & Wensel, T. G. (1995). *Inorg. Chem.* **34**, 864–869.

[bb16] Liu, Y., Dou, J., Wang, D., Li, D. & Gao, Z. (2006). *J. Chem. Crystallogr.* **36**, 613–618.

[bb17] Lukes, R. & Jurecek, M. (1948). *Collect. Czech. Chem. Commun.* **13**, 131–160.

[bb18] Rigaku/MSC (2006). *CrystalClear* Version 1.3. Rigaku/MSC, The Woodlands, Texas, USA.

[bb19] Su, H., Wen, Y. H. & Feng, Y. L. (2005). *Z. Kristallogr. New Cryst. Struct.* **220**, 560–562.

[bb20] Yang, L., Crans, D. C., Miller, S. M., la Cour, A., Anderson, O. P., Kaszynski, P. M., Godzala, M. E., Austin, L. D. & Willsky, G. R. (2002). *Inorg. Chem.* **41**, 4859–4871. 10.1021/ic020062l 12230390

[bb21] Zhou, G.-W., Guo, G.-C., Liu, B., Wang, M.-S., Cai, L.-Z. & Huang, J.-S. (2004). *Bull. Korean Chem. Soc.* **25**, 676-680.

